# 2024 Egyptian consensus statement on the role of non-statin therapies for LDL cholesterol lowering in different patient risk categories

**DOI:** 10.1186/s43044-024-00562-7

**Published:** 2024-09-20

**Authors:** Hesham Salah El Din Taha, Hossam Kandil, Hala Mahfouz Badran, Nabil Farag, Hazem Khamis, Gamila Nasr, Mina Samy, Moustafa Abdrabou, Mohamed Abuelezz, Mirna Mamdouh Shaker

**Affiliations:** 1 Faculty of Medicine, Cairo University, Cairo, Egypt; 2 Faculty of Medicine, Menoufia University, Shebin El Kom, Egypt; 3 Faculty of Medicine, Ain Shams University, Cairo, Egypt; 4 Wadi Elneel Hospital, Cairo, Egypt; 5 Faculty of Medicine, Suez Canal University, Ismailia, Egypt

**Keywords:** Non-statin therapies, LDL cholesterol lowering, Patient risk categories

## Abstract

**Background:**

The new millennium has witnessed increased understanding of cardiovascular (CV) risk factors and improvement in atherosclerotic cardiovascular disease (ASCVD) management. The role of LDL cholesterol and other atherogenic lipid particles in the development of atherosclerosis is now beyond doubt.

**Main body:**

Statins have been widely used and recommended in guidelines for preventing and managing ischemic events. However, statins have side effects, and many patients do not achieve their low-density lipoprotein cholesterol (LDL-C) goals. In recent years, non-statin lipid-lowering agents have gained increasing use as adjuncts to statins or as alternatives in patients who cannot tolerate statins. This consensus proposes a simple approach for initiating non-statin lipid-lowering therapy and provides evidence-based recommendations. Our key advancements include the identification of patients at extreme risk for CV events, the consideration of initial combination therapy of statin and ezetimibe in very high-risk and extreme-risk groups and the extended use of bempedoic acid in patients not reaching LDL-C targets especially in resource-limited settings.

**Conclusions:**

Overall, this consensus statement provides valuable insights into the expanding field of non-statin therapies and offers practical recommendations to enhance CV care, specifically focusing on improving LDL-C control in Egypt. While these recommendations hold promise, further research and real-world data are needed for validation and refinement.

## Background

Cardiovascular diseases (CVD) have been a leading cause of death and disability worldwide for many years 1. In 2021, the Global Burden of Disease (GBD) study linked elevated low-density lipoprotein cholesterol (LDL-C) levels to 3.81 million deaths from CVD and other causes. 2

Statins have long been the first option for LDL-C reduction and have led to a revolutionary improvement in CV outcomes, particularly for high-risk patients. Meta-analyses of randomized clinical trials showed that 1 mmol/L reduction in LDL-C cardiovascular (CV) risk by 21%. 3

However, studies have shown that many patients fail to achieve the recommended LDL-C goal, despite being on high-intensity statins [4–8. This suboptimal response to statins can be due to various factors, including genetic factors, statin intolerance and physician inertia [9–12. Patients with familial hypercholesterolemia (FH) often require additional therapies beyond high-intensity statins to effectively lower LDL-C levels. 13

Challenges such as incomplete adherence, statin intolerance and long-term risks faced by those with inadequate LDL-C control have led to a growing emphasis on the development of alternative forms of lipid-lowering therapy (LLT) as an adjunct or alternative to statins. Furthermore, there is growing interest in early combination lipid-lowering management approaches 14, as they offer several benefits, such as incremental LDL-C lowering, reduced risk for side effects and improved tolerability and adherence 15, 16. In the DYSIS-EGYPT study, almost one-third of patients (30%) were receiving other lipid-lowering therapies, primarily ezetimibe (23.7%), but also fibrates (8.3%), bile acid sequestrants (1.0%) and nicotinic acid (0.3%). However, the percent of patients reaching LDL-C goals was overall low 17. The evolving landscape of non-statin therapies will continue to provide patients with more therapeutic options for reducing CV risk, each with unique indications, advantages, disadvantages and evidence base. 18, 19

Despite evidence of the effectiveness of proprotein convertase subtilisin/kexin type 9 inhibitors (PCSK9-I), a large multicenter project revealed that they were prescribed to less than 1% of over 3.5 million patients 20. This low prescription rate can be attributed to factors such as cost, initial lack of outcome data, prior authorization requirements and lack of insurance approval 21. Ultimately, the high cost of treatment limits the use of PCSK9-I. 22

Egypt is classified as a country with a very high CV risk, with CVD accounting for 46.2% of non-communicable disease-related deaths in 2017 23. The Egyptian CardioRisk project published in 2020 found that 51% of Egyptians had premature acute coronary syndrome (ACS) 24, 25, and dyslipidemia was a prevalent modifiable risk factor, affecting 48.2% of individuals 26. Given the limited resources in Egypt, alternative approaches are necessary to reduce CV risk and identify specific high-risk populations that can benefit from costly medications.

## Main text

### Rationale for use of non-statins

The response to statins and the achievement of LDL-C goals in primary and secondary prevention patients have been the subject of several recent studies [4–8. The TERESA study revealed that only 31.1% of patients achieved the risk-based LDL-C goals, despite being on high-intensity statins, with nearly 70% of very high-risk patients failing to reach the recommended LDL-C goals 8. Statin hyporesponsiveness can be attributed to various factors, including rare genetic variants in lipoprotein-related or drug metabolism genes 9, 10. Before considering primary statin resistance, other potential causes such as analytical issues with LDL-C measurement and the presence of common lipid disorders [FH, elevated lipoprotein (a) (Lp(a)) and secondary dyslipidemias] should be ruled out. 13

Nonetheless, the most common reason for suboptimal response to statin therapy is the lack of compliance due to statin intolerance. The lipid management registry showed that over 50% of former statin users discontinued the medication due to perceived side effects, primarily muscle-related symptoms 11. Similarly, a recent meta-analysis estimated that between 5 and 17% of patients discontinue statins due to medication side effects, which is significantly higher than rates observed in clinical trials. 12

The SANTORINI study assessed lipid management in 9044 patients after the 2019 ESC/EAS guidelines update. It found that 80% of high- and very high-risk patients did not meet LDL-C goals. 27

In the DA VINCI study which involved 5888 patients, 54% of patients achieved the 2016 risk-based goal, while 33% achieved the 2019 goal. High-intensity statin monotherapy was used in 20% of very high-risk primary prevention patients and 38% of secondary prevention patients. Combination therapies, including ezetimibe (9%) or PCSK9-I (1%), were used less frequently. The corresponding 2016 and 2019 goal attainment was 53% and 20% for moderate–high-intensity statin combination with ezetimibe, and 67% and 58% for PCSK9-I combination. 28

Therefore, there are persistent gaps between clinical guidelines and clinical practice for lipid management across the world. To address these gaps, greater utilization of non-statin LLT even with optimized statins, especially for patients at the highest risk, is needed.

In recent years, clinical trials have demonstrated the efficacy of several non-statin agents in lowering LDL-C levels and reducing CV events in specific patient populations [29–47. Landmark trials have established ezetimibe, PCSK9 monoclonal antibodies and bempedoic acid as non-statin agents that lower LDL-C levels and provide CV benefits. Additionally, a large-scale outcome trial is underway to evaluate the efficacy of inclisiran. 48

There is growing interest in early combination lipid-lowering management approaches 14, as they offer several benefits such as incremental LDL-C lowering, reduced risk for side effects and improved tolerability and adherence 15, 16. Combination therapies, especially including a PCSK9-I, have been shown to achieve the recommended LDL-C goals in a higher percentage of very high-risk patients compared to high-intensity monotherapy or statin/ezetimibe combination. 28

### Cardiovascular risk stratification

Total CV risk is defined as the likelihood of a person to develop an atherosclerotic CV event within a given time period. It is determined by adding the risk contribution of various CV risk factors present in an individual. Some risk factors, such as a history of a previous atherosclerotic event, represent a very high total CV risk on their own, even in the absence of other risk factors.

There is no universal consensus on the definition of very high-risk patients, but it is recommended to intensify preventive approaches for these patients in all guidelines. The very high-risk patient category definition is different between guidelines. All guidelines base the intensity of their recommendations on the degree of risk. Guidelines differ in their risk calculation systems.

In the American Heart Association (AHA)/American College of Cardiology (ACC)/Multisociety (MS) guidelines, risk scores are calculated with pooled cohort equations (PCEs). PCEs calculate the 10-year risk of developing ASCVD by including non-fatal myocardial infarction (MI) or coronary artery disease (CAD) death and fatal or non-fatal stroke, among people free from ASCVD 1. In the ACC Expert Consensus Decision Pathway published in 2022 on the use of non-statins, patients with ASCVD were categorized into one of two groups: not at very high risk or at very high risk. Very high-risk patients have a history of multiple major ASCVD events or one major ASCVD event and multiple high-risk conditions. Major ASCVD events include ACS within 12 months, history of MI, ischemic stroke or symptomatic peripheral arterial disease (PAD). High-risk conditions include age ≥ 65 years, heterozygous FH (HeFH), history of prior coronary artery bypass surgery (CABG) or percutaneous coronary intervention (PCI) outside of the major ASCVD event(s), diabetes mellitus (DM), hypertension (HTN), chronic kidney disease (CKD) (eGFR 15–59 mL/min/1.73 m^2^), current smoking, persistently elevated LDL-C ≥ 100 mg/dL despite maximally tolerated statin therapy and ezetimibe or history of congestive heart failure (CHF). 2

The European Society of Cardiology (ESC) guidelines described low-, intermediate-, high- and very high-risk categories of ASCVD risk. People were sorted into one of these categories depending on the presence or absence of major risk factors and, in apparently healthy persons, depending mainly on their 10-year CV risk estimation. 49, 50

The ESC guidelines define patients with ASCVD, DM, CKD and individuals with specific risk factors as high- and very high-risk groups automatically. Individuals who do not have these characteristics are considered as apparently healthy people, and management is determined according to risk estimation by the SCORE2 and the SCORE2-Older Persons (SCORE2-OP) model for adults over the age of 69, which calculate the 10-year risk of total CV events. In diabetic patients, CV risk stratification is based on ASCVD, severe target organ damage (TOD) or SCORE2-Diabetes. Management is determined according to age, risk score and region. 49, 50

Ray et al. defined the extremely high-risk patient category as having one of the following characteristics: post-ACS and a history of other vascular event in the past 2 years, PAD, polyvascular disease, multivessel CAD and/or FH. 14

The American guidelines give recommendations for the possible use of the coronary artery calcium (CAC) score if the decision about statin treatment is uncertain in intermediate- and borderline-risk adults. If the CAC score is above 100, it is reasonable to initiate statin treatment. If the CAC score is 1–99, it is reasonable to use statins in individuals > 55 years. If the CAC score is zero, there is no need to use statins as long as higher-risk conditions are absent, but reassessment is suggested in 5–10 years 29. Similarly, the ESC guideline states that CAC scoring may be considered to improve risk classification around treatment decision thresholds, and they also consider plaque detection by carotid ultrasound an alternative when CAC scoring is unavailable or not feasible. 51

**Fig. 1:**
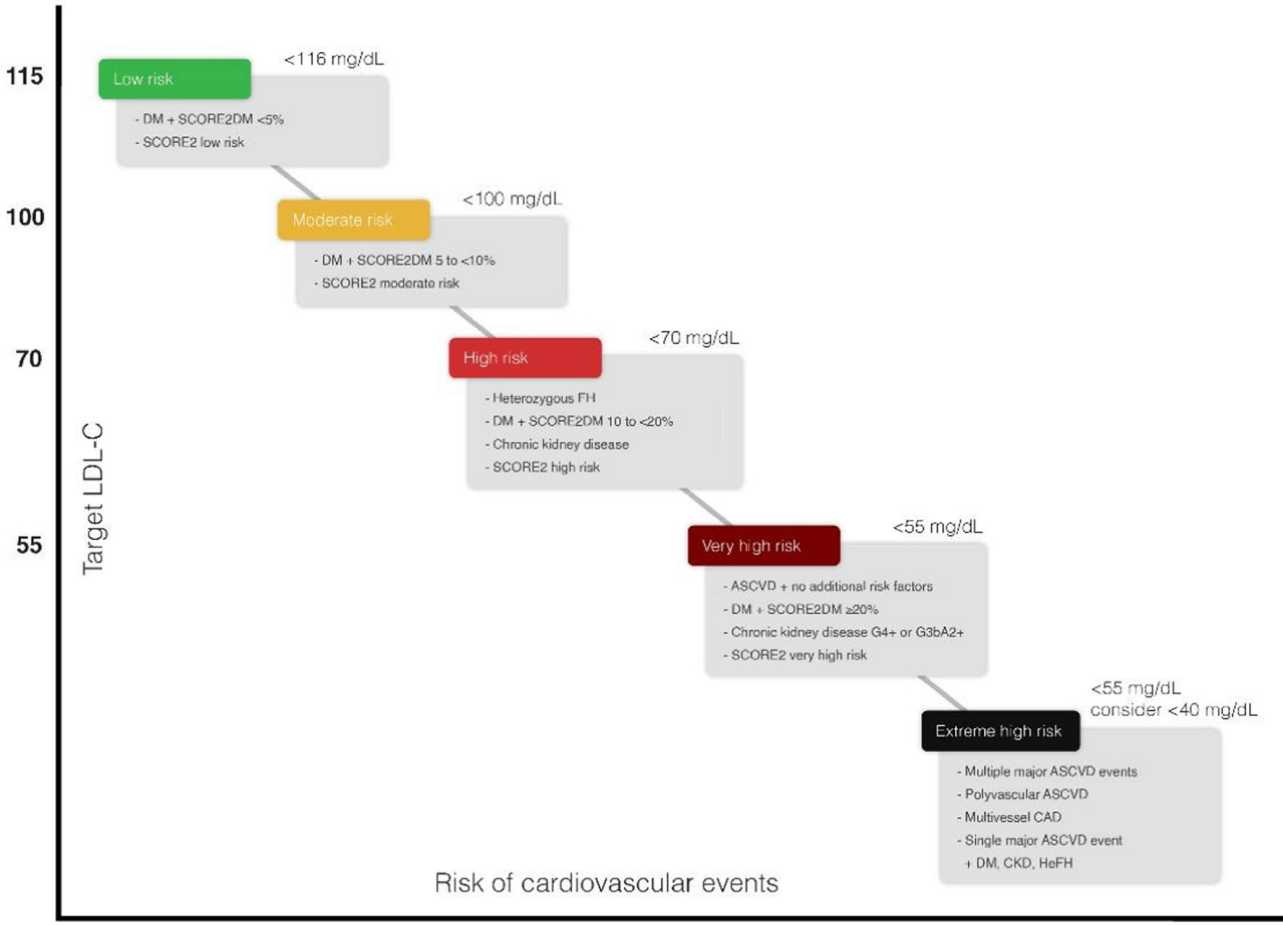
Patient risk categories and recommended LDL-C goals

We endorse the CV risk stratification recommended by the latest ESC guidelines 49, 50. However, we recommend distinguishing patients who are at extremely high CV risk from the very high-risk category. We propose defining the extreme-risk patient category by the presence of at least one of the following criteria: (1) multiple major CV events, especially if recurrent within 2 years, (2) one major CV event and multiple high-risk conditions such as current smoking, DM, HTN and CKD, (3) polyvascular disease, (4) multivessel CAD and (5) recent ACS within the past 12 months (Fig. 1).

For patients in the low- to moderate- and high-risk conditions, it is advisable to check for risk modifiers. The presence of these risk modifiers upgrades the patient’s risk category and supports the use of LLT. Risk modifiers include family history of premature ASCVD (men aged < 55 years; and women aged < 65 years), familial dyslipidemia, metabolic syndrome, CKD, chronic immune-mediated inflammatory conditions, sex-specific conditions (history of premature menopause before age 40 years, pregnancy-related HTN and erectile dysfunction), psychosocial factors, left ventricular hypertrophy, atrial fibrillation, obstructive sleep apnea syndrome, non-alcoholic fatty liver disease, migraine with aura and if measured, CAC score > 0 Agatston units (AUs), elevated high-sensitivity C-reactive protein (> 2.0 mg/L), high Lp (a) > 50 mg/dL and ankle brachial index (ABI) < 0.9.

### Lipid-lowering strategies

Statins, or hydroxymethylglutaryl-coenzyme A (HMG-CoA) reductase inhibitors, are widely prescribed and effective in lowering cholesterol levels by inhibiting the enzyme responsible for cholesterol production. However, some patients experience side effects, such as muscle pain or liver abnormalities, leading to discontinuation or reduced adherence. 52

### ***Non-statin options ***(Fig. 2)

**Fig. 2:**
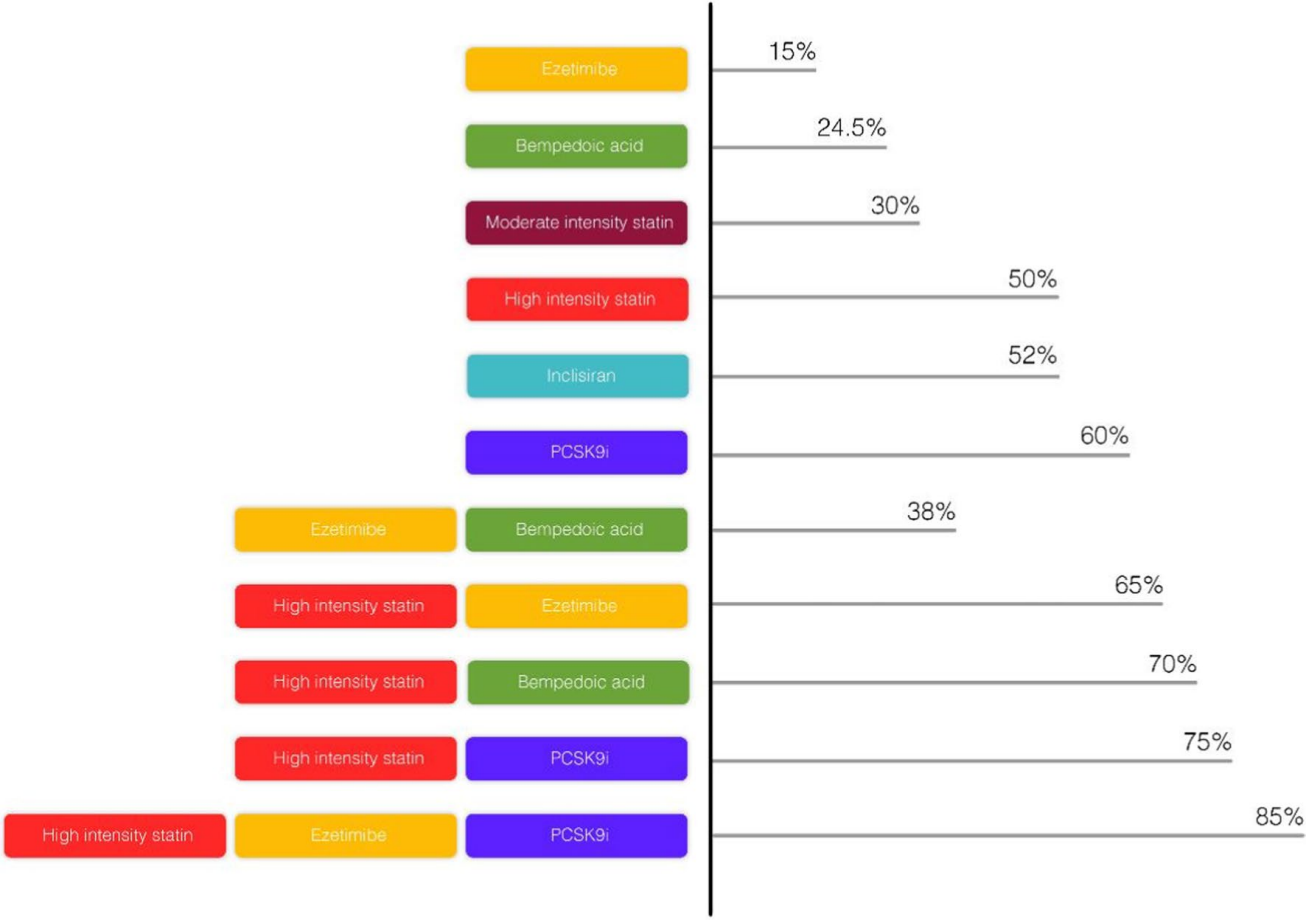
Efficacy of different lipid-lowering medications as monotherapy or in combination

1. **Ezetimibe**
  Ezetimibe inhibits cholesterol absorption at the brush border of the small intestine by binding to the sterol transporter Niemann–Pick C1-Like-1 (NPC1L1). The reduction in cholesterol absorption, when combined with statins, offers a synergistic effect on LDL-C levels lowering. 53
  Ezetimibe, given as a monotherapy in a single daily oral dose of 10 mg, reduces LDL-C levels by 15–22% compared to placebo. Adding ezetimibe to statin therapy reduces LDL-C by an additional 21–27%. Ezetimibe decreased triglyceride (TG) by about 8% and increased high-density lipoprotein cholesterol (HDL-C) by about 5%. 53
  The IMPROVE-IT trial evaluated the effect of ezetimibe and simvastatin compared with simvastatin alone in patients with ACS. This study which included over 18,000 patients with ACS found that adding ezetimibe to statin therapy lowered LDL-C by 24% and the major adverse CV events (MACE) by 2% (absolute risk difference). 54
  The US Food and Drug Administration (FDA) gave a category C mark to ezetimibe based on preclinical data from animal studies; however, no enough data is available to support the use of ezetimibe in pregnancy and lactation. The use of ezetimibe in children is not studied. 55
  The most frequent side effects of ezetimibe are diarrhea (4%) and headache (2%) while liver injury is extremely rare. Ezetimibe use is contraindicated in moderate and severe liver impairment. There is no need for dose modification in renal impairment or mild hepatic impairment. 54
2. **PCSK9-targeted therapies**
  PCSK9 is a protease that degrades LDL receptors in the liver. Human monoclonal antibodies, alirocumab and evolocumab, administered through subcutaneous injections bind to PCSK9, preventing it from binding to LDL receptors. Inclisiran is a synthetic small interfering RNA (siRNA) that targets hepatic production of PCSK9. By preventing the destruction of the LDL receptors, these drugs enhance the ability of the liver to clear LDL-C from the bloodstream significantly reducing its level. 56
  Multiple trials have demonstrated not only the potency of PCSK9-I in significantly lowering LDL-C levels 56, but also their efficacy in improving CV outcomes with reduction in MACE, including heart attacks and strokes.
  In 2017, the FOURIER trial demonstrated that inhibition of PCSK9 with evolocumab on a background of statin therapy was efficacious in patients with stable ASCVD and additional high-risk features. The ODYSSEY Outcomes (Evaluation of Cardiovascular Outcomes After an Acute Coronary Syndrome During Treatment With Alirocumab) trial demonstrating the benefits of PCSK9 inhibition with alirocumab on a background of statin therapy in patients with ACS. 31, 57
  In a pooled patient-level analysis of 3660 patients in phase 3 trials of inclisiran (ORION-9, ORION-10 and ORION-11), the drug demonstrated a mean placebo-corrected change in LDL-C at day 510 of 50.7% (95% CI 52.9% to 48.4%; *P* < 0.0001). The corresponding time-adjusted mean change in LDL-C was 50.5% (95% CI 52.1% to 48.9%; *P* < 0.0001) 58. Inclisiran was FDA approved in December 2021 and is indicated as an adjunct to diet and maximally tolerated statin therapy for the treatment of adults with HeFH or ASCVD who require additional lowering of LDL-C 59. The recommended dosage of inclisiran, in combination with maximally tolerated statin therapy, is 284 mg administered as a single subcutaneous injection initially, again at 3 months, and then every 6 months thereafter. The effect of inclisiran on CV morbidity and mortality has not been determined. However, we are waiting the results of two important CV outcomes trials, ORION-4 (A Randomized Trial Assessing the Effects of Inclisiran on Clinical Outcomes Among People With Cardiovascular Disease) and VICTORION-2P (A Randomized, Double-blind, Placebo-controlled, Multicenter Trial, Assessing the Impact of Inclisiran on Major Adverse Cardiovascular Events in Participants With Established Cardiovascular Disease), which are currently in progress. 59
  Generally, PCSK9-I has demonstrated a favorable safety profile. However, like any medication, they may be associated with certain side effects, including injection-site reactions.
  The cost of the PCSK9-I remains a major obstacle in the large-scale use of these drugs. Another challenge is the need for subcutaneous injections. Efforts to address these challenges and ongoing research into alternative administration methods may further broaden the accessibility and utilization of these inhibitors. 60
3. **Bempedoic acid**
  Bempedoic acid is a prodrug that is activated in the liver. It competitively inhibits ATP citrate lyase (ACL), an enzyme that operates upstream of HMG-CoA reductase. This allows bempedoic acid to effectively lower LDL-C levels.
  Bempedoic acid and the fixed-dose combination with ezetimibe were FDA approved in 2020 and are indicated as an adjunct to diet and maximally tolerated statin therapy for the treatment of adults with HeFH or ASCVD who require additional lowering of LDL-C. 61
  The daily dose is 180 mg, administered orally, once daily. No renal or hepatic dose adjustment is required for mild or moderate renal or hepatic impairment. Bempedoic acid is not studied in severe renal impairment (eGFR < 30 ml /min/m^2^) and severe hepatic impairment (Child–Pugh C). No data is available on the use of bempedoic acid in pregnancy or lactation in humans. However, no teratogenicity was detected in animal studies.
  In the clear outcome trial, involving almost 14,000 patients for primary or secondary prevention, bempedoic acid significantly reduced LDL-C levels by 18%. This reduction in LDL-C levels translated into a 21% lower risk of MACE, including heart attack, stroke and CV death. Bempedoic acid also lowers hs-CRP; however, it does not significantly affect TG and Lp(a) levels. 62
  Bempedoic acid has a favorable safety profile. The most common side effects include hyperuricemia, nasopharyngitis and upper respiratory tract infection. The rare occasion of tendon rupture, in clear outcome trial, was non-significant; however, bempedoic acid should be discontinued immediately if the patient experiences tendon rupture. Consideration of an alternative therapy is advised in patients with a history of tendon disorders or tendon rupture. Muscle-related side effects are usually related to the concomitant use of high-intensity statin, and this goes in favor of the use of bempedoic acid in statin intolerance. 62
  Bempedoic acid offers a valuable addition to the available cholesterol-lowering therapies. It provides an effective, relatively cheap and well-tolerated option for patients with high LDL-C levels who cannot tolerate statins or require additional LDL-C lowering beyond what statins can provide. 62
4. **Fibrates**
  Fibrates, such as fenofibrate, primarily target TG levels and raise HDL-C. They are often considered in patients with hypertriglyceridemia or mixed dyslipidemia. Fibrates may also be used in combination with statins for comprehensive lipid management. The positive effects fenofibrate on diabetic retinopathy may have important clinical implications. The Accord-Eye study, using fenofibrate combined with statin, demonstrated a reduced progression of diabetic retinopathy. 63
5. **Bile acid sequestrants**
  Bile acid sequestrants bind to bile acids in the intestine, preventing their reabsorption. This process enhances the clearance of LDL-C from the blood. While effective, these agents may be associated with gastrointestinal side effects, impacting their tolerability. 64
6. **Evinacumab**
  Angiopoietin-like protein 3 (ANGPTL3) pharmacologic inhibition reduces LDL-C levels independently of the LDL receptor. Evinacumab, a human monoclonal antibody that inhibits of ANGPTL3, was initially evaluated in patients with homozygous FH (HoFH), who may have absent or defective LDL receptors 65. In 2021, evinacumab received FDA approval and is indicated as an adjunct to other LDL-C–lowering therapies for the treatment of adult and pediatric patients, aged 12 years and older, with HoFH 66. Evinacumab is also being investigated for the treatment of patients with severe hypertriglyceridemia.
7. **Lomitapide and mipomersen**
  Lomitapide is a selective inhibitor of the microsomal triglyceride transfer protein (MTP) and reduces LDL-C levels by approximately 40%. It may cause elevations in liver transaminases and hepatic steatosis with the risk for progressive liver disease. 67
  Mipomersen is an antisense oligonucleotide that inhibits the production of apoB-100 and lowers LDL-C by approximately 37%. Both medications represent new therapeutic approaches for patients with HoFH who do not reach LDL targets with statins. 67

### LDL apheresis

It selectively removes apo B-containing lipoproteins, producing an acute reduction in LDL-C. The FDA-approved indications include: patients with FH unresponsive to pharmacologic and dietary management who are either functional homozygotes with an LDL-C > 500 mg/dL, functional heterozygotes with no known CV disease but an LDL-C > 300 mg/dL or functional heterozygotes with known CVD and LDL-C > 200 mg/dL 68. The extracorporeal technique is performed weekly or biweekly with an average LDL-C reduction of ∼50–60% of the original values. Adverse effects include problems with venous access; transient hypotension and bleeding. CV outcomes trials are limited due to lack of randomized controlled trials of these very high-risk patients. 69

### Approach to LDL-C lowering according to patient risk category

According to the 2019 ESC guidelines, all patients in the very high-risk category should be managed similarly with a target LDL-C goal of below 55 mg/dL. Only patients with recurrent major ASCVD events within 2 years, despite optimal risk reduction measures, should be considered for a lower LDL-C target of below 40 mg/dL, as a class IIb recommendation 5. On the other hand, the 2022 ACC Expert Consensus Decision Pathway sets the LDL-C goal for high-risk patients with clinical ASCVD at below 70 mg/dL and for those at very high risk at below 55 mg/dL.

However, evidence from the FOURIER 6 and ODYSSEY 7 trials suggest that patients at extreme CV risk may benefit from even lower lipid targets and more aggressive CV risk reduction measures compared to the rest of the very high-risk category. These patients may also benefit from the use of combination therapy as an initial treatment approach. Ray et al. encouraged this approach advocating the idea of the use of triple initial therapy in the form of statins, ezetimibe and PCSK9-targeted therapy in the extremely high-risk cohort. 14

The WHO classified the Egyptian population as having a very high risk of CVD mortality 23. The DYSIS study revealed that the achievement of LDL-C goals in the Egyptian cohort was lower compared to other populations, with only 33% of patients meeting the target. The above-target LDL-C levels were found in only 28% of high- and very high-risk patients and 50% of moderate-risk patients 17. This highlights the need for amended strategies to address poor LDL-C management in Egypt and encourage a lower LDL-C therapeutic goal.

Based on the data presented, we have formulated the following recommendations for ASCVD risk management. Firstly, it is crucial to distinguish individuals at extremely high ASCVD risk from the rest of the very high-risk category. For these individuals, we suggest initiating combination therapy with a high-intensity statin, if tolerated, and ezetimibe as an initial approach. The LDL-C target for this group should be below 55 mg/dL, with consideration of a lower initial target of below 40 mg/dL on an individual basis. Initial combination therapy with ezetimibe may also be considered in the very high-risk category when a 50% reduction in LDL-C is not expected to get the patient to target. For other risk categories, we advise following the recommendations outlined in the ESC dyslipidemia guidelines.

If additional LDL-C lowering of less than 20–30% is required, particularly in the non-acute setting, the addition of bempedoic acid may be considered. However, if a greater reduction in LDL-C is necessary for individuals in very high- or extreme-risk categories, PCSK9-targeted therapy should be considered. Although there have been no large-scale studies comparing PCSK9-I and bempedoic acid to date, both medications have been studied in thousands of patients with various risk profiles and have each demonstrated the ability to achieve their primary hard endpoints, resulting in a significant reduction in CV morbidity and mortality. It is also noteworthy that, while not yet included in the guidelines, bempedoic acid can serve as an option for optimizing lipid control in resource-limited settings.

**Fig. 3:**
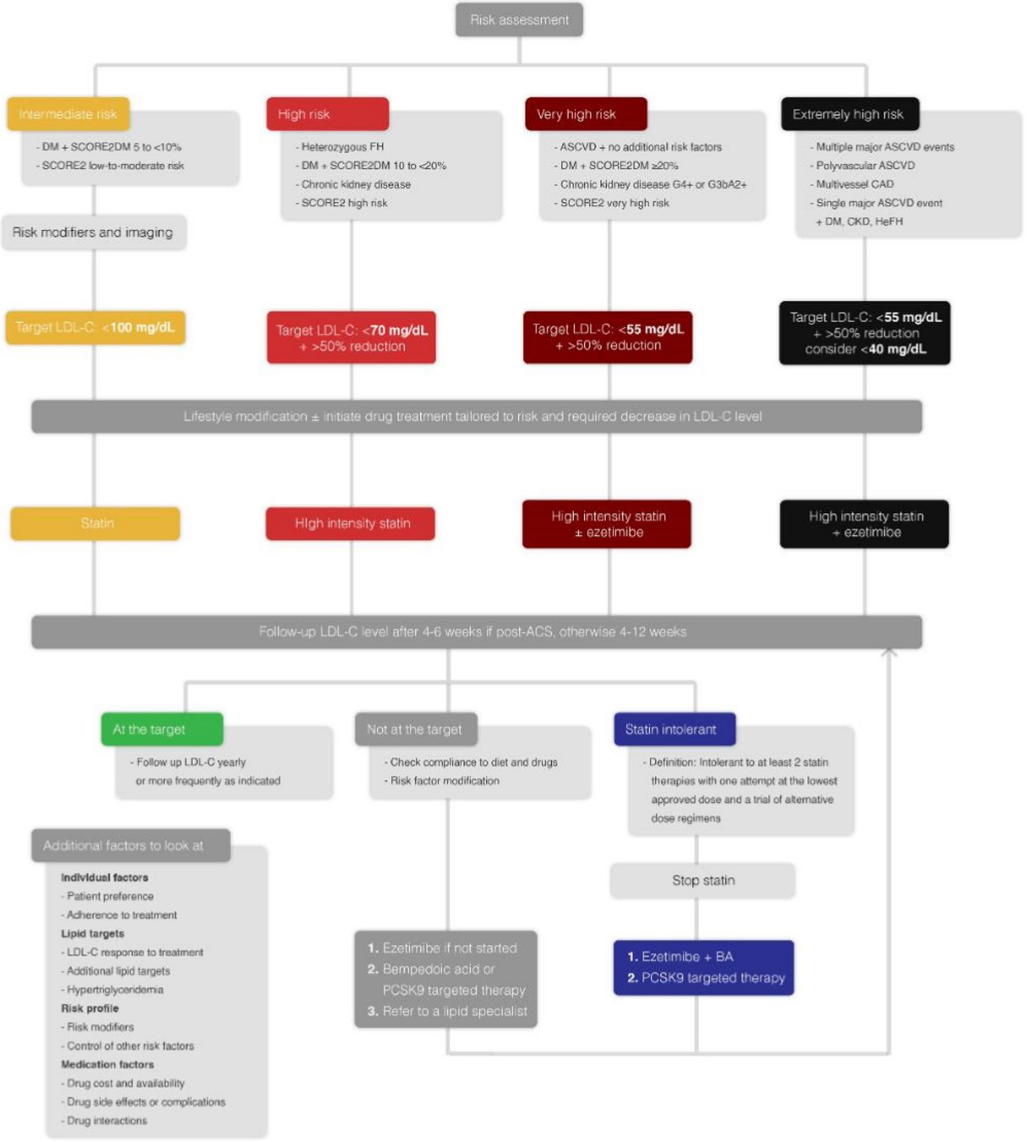
Risk categories, LDL-C goals and suggested management strategies

In patients who are elderly, at risk of myopathy, having CKD or having partial statin intolerance, we advise to consider initiation of LLT with a combination of moderate-intensity statin and ezetimibe. In case the statin intolerance is complete, an initial combination of ezetimibe and bempedoic acid can be considered. Further addition of PCSK9-targeted therapy may be needed according to the LDL-C level achieved. Finally, the clinician’s selection of the appropriate therapy requires discussion and a shared decision-making with the patient to ensure personalized and effective management of ASCVD risk (Fig. 3).

### Monitoring of response to lipid-lowering therapy

Both the European and American guidelines recommend assessing the response to pharmacological therapy at 4–12 weeks from initiation or adjustment of therapy. This initial follow-up allows for the evaluation of treatment effectiveness and any necessary adjustments. Subsequent follow-up intervals after the LDL-C goal is attained differ between guidelines. The European guidelines suggest 6–12 months for monitoring, while the American guidelines recommend 3–12 months. 29, 51

The ESC guidelines recommend shorter initial follow-up intervals of 4–6 weeks after ACS to ensure early and proper LDL-C goal achievement 51. This recommendation is based on the need for closer monitoring during the early stages of recovery from the acute event. Our writing committee also advocates comparable initial follow-up intervals for patients at extreme risk, aiming to optimize care and minimize risks. Additionally, we recommend subsequent follow-up intervals of 6 months for this particular patient risk category, ensuring ongoing care and risk reduction and earlier if dose adjustments were done.

During follow-up, a full lipid profile should be performed. Additionally, non-HDL-C or ApoB should also be analyzed and used as a secondary treatment target. Adherence to both medication and lifestyle regimens is crucial for maximizing the reduction of ASCVD risk. 29, 51

### Non-statin uses in special populations

#### Diabetes

The use of statins in diabetic patients in the primary prevention trials has shown significant CV outcome benefit, thus all diabetic patients aged from 40 to 75, and having no contraindications, will benefit from at least moderate-intensity statins, whereas higher-risk diabetic patients (e.g., older patients, patients with additional risk factors, albuminuria, retinopathy, long-standing DM, CKD or any sort of diabetic nephropathy) would probably benefit from high-intensity statins.

The target LDL level depends upon the risk stratification of the patient based on ASCVD, severe TOD or SCORE2-Diabetes. The first line should be statins; however, if the target LDL was not achieved on the maximally tolerated statin dose, the physician should share a decision-making involving the patient on using another non-statin therapy.

The initial non-statin therapy should be ezetimibe 10 mg daily dose. According to the writing committee, bempedoic acid and/or PCSK9-targeted therapy can be considered if patients have an inadequate response to statin/ezetimibe or if patients are statin-intolerant. Inclisiran has currently no established evidence-based role for primary prevention of ASCVD in patients with diabetes; however, it can be considered if patients do not reach their targets on statin/ezetimibe combination in very high- or extreme-risk patients. 70

### Chronic kidney disease

Although many of the initial statin CVD studies did not include many CKD patients, evidence from subgroup analyses of large statin studies suggested that CKD subjects had similar benefits to non-CKD individuals 13, 14. Furthermore, for CKD patients with pre-end-stage renal disease, statins effectively lower total cholesterol and LDL cholesterol levels and decrease CVD risk. However, the benefit of statins is unclear in patients with nephrotic syndrome and was absent in patients with only microalbuminuria 15 and those on regular hemodialysis.

Fibrates are known to decrease renal blood flow and glomerular filtration, and they are cleared renally; therefore, there is significant concern regarding their use in CKD.

Ezetimibe is metabolized through intestinal and hepatic metabolism, and does not require any dose adjustment in CKD or ESRD, making it potentially attractive therapy in CKD.

A secondary analysis from the IMPROVE IT trial evaluating outcomes based on eGFR shows that compared to statin alone, the combination of statin + ezetimibe was more effective in reducing risk of CVD outcomes in those with eGFR < 60/ml/min/1.73 m^2^. 16

The effects of evolocumab treatment on LDL lowering were similar in the normal renal function group and the renally impaired groups, with no clinically important adverse events 18. Regarding alirocumab, a subgroup analysis from Odyssey Outcomes Trial, showed it to be safe down to GFR of 30, but for GFR less than 30, there are no available data. 19

Bempedoic acid use in CKD is approved without dosage adjustment for eGFR > 30 ml/minute/1.73 m^2^. As bempedoic acid has hepatic metabolism, it is presumably safe in CKD.

Regarding inclisiran, there is no recommended dosage adjustment in CKD, but there have been no studies done in patients with ESRD. An analysis of the ORION-1 and ORION-7 studies compared inclisiran in patients with renal impairment and those with normal renal function, and found similar safety and efficacy, suggesting that no dose adjustment is needed in CKD. However, no patients on dialysis were studied in these trials.

### Heart failure

#### Statin use in HF

The use of statins in patients with symptomatic heart failure with reduced EF was addressed in two trials (CORONA and GISSI-HF), both trials did not show significant reductions in primary endpoints; however, a subsequent meta-analysis of these trials demonstrated a significant 19% reduction in MI rates among patients with ischemic etiology of heart failure; therefore, the writing committee of this consensus states that it is reasonable to consider the use of statins in patients with symptomatic heart failure due to ischemic etiology or in the presence of dyslipidemia necessitating treatment. [71–73

#### Ezetimibe use in HF

A post hoc analysis of IMPROVE-IT predicted the greatest likelihood from adding of ezetimibe to statin therapy following ACS in heart failure subgroup 42; therefore, the writing committee of this consensus recommends that for patients with ASCVD and a history of heart failure who achieve inadequate lowering of LDL-C on maximally tolerated statin therapy, the addition of ezetimibe may be reasonable.

#### PCSK9 use in HF

Alirocumab failed to reduce MACE in HF patients in a post hoc analysis and was associated with increase in non-fatal MI. Based on this, the writing committee of this consensus judges that no recommendations can be made in this regard, and the patients with ASCVD and NYHA II–III HF due to ischemic etiology should follow the algorithm for patients with ASCVD at very high-risk on statin therapy for secondary prevention.

#### Inclisiran and bempedoic acid use in HF

Inclisiran trials 74 have excluded patients with HF NYHA classes II, III, and IV and patients with LVEF < 30%. Also, the CLEAR-OUTCOMES trial of bempedoic acid excluded patients with severe heart failure. Therefore, the writing committee of this consensus judges that no recommendations can be made in this regard.

### Women considering pregnancy or already pregnant

#### Statin use

Until 2021, the use of statins in pregnancy was absolutely contraindicated. However, in July 2021, the FDA requested revisions to the information regarding use of statins in pregnancy, removing the contraindication against use in all pregnant patients 75. However, the writing committee of this consensus does not recommend the use of statins in pregnant or breastfeeding mothers pending further safety information. Referral to a lipid specialist for high-risk patients including homozygous familial hypercholesterolemia and those who have clinical ASCVD is advised. We also recommend to stop statins for at least 2 months before planned pregnancy and to refer those at elevated risk to a lipid specialist.

#### Ezetimibe/bempedoic acid/inclisiran/PCSK9

There are no well-controlled trials for the use of these drugs during pregnancy; therefore, the writing committee of this consensus stated that no recommendation can be made in this regard.

### Elderly

Few studies have focused on lowering LDL-C in elderly patients (≥ 75 years)**.** The PROSPER Trial investigated the effect of pravastatin (*n* = 2891) versus placebo (*n* = 2913) on CV events in older subjects, statins lowered LDL-C by 34% compared to the placebo group and reduced the primary end point by 15% in patients with preexisting CVD. However, the study failed to demonstrate the benefit of statins in the elderly without CVD 76. According to the writing committee, the initial non-statin therapy should be ezetimibe 10 mg daily dose. Bempedoic acid and/or PCSK9-targeted therapy can be considered if patients have an inadequate response to statin/ezetimibe or if patients are statin-intolerant.

### Familial hypercholesterolemia

The previous studies have reported that only a minority of the FH patients reached their LDL-C target. The main determinants associated with reaching the LDL-C target were a lower LDL-C before treatment and treatment with a PCSK9-I. 77

In HeFH, both alirocumab and evolocumab have been tested. In the Rutherford-2 trial, evolocumab lowered LDL-C by 60%, non-HDL-C by 56%, apolipoprotein B by 49%, Lp(a) by 31% and triglycerides by 22% while increasing HDL by 8% 78. In the Odyssey FH I and FH II studies, alirocumab lowered LDL-C by approximately 55%, non-HDL-C by ~ 50%, apolipoprotein B by ~ 43%, Lp(a) by ~ 19% and TG by ~ 14% while increasing HDL by ~ 7% 79. Thus, in these difficult to treat patients, PCSK9 monoclonal antibodies were still very effective at lowering pro-atherogenic lipoproteins.

In HoFH, evolocumab resulted in a 21–31% decrease in LDL-C compared to placebo in patients with homozygous FH 80, 81. The response to treatment seems to be dependent on the primary genetic cause. Patients with mutations in the LDL receptor leading to the expression of defective receptors respond to therapy whereas patients with mutations leading to negative receptors (null variants) have a poor response. The mechanism by which PCSK9 inhibitors lower LDL-C levels is not surprising, as patients that do not have any functional LDL receptors will not respond to therapy. Alirocumab decreased LDL-C by 35.6%, non-HDL-C by 32.9%, apolipoprotein B by 29.8% and Lp (a) by 28.4% (84,85). Given that PCSK9 monoclonal antibodies decrease LDL-C levels in some FH patients, these drugs can be useful in this very difficult-to-treat patient population. [80–83

### Patients with statin-associated side effects

The most-encountered statin-associated side effects (SASEs) in clinical practice is statin-associated muscle symptoms, which may occur in 5–20% of patients; however, true complete intolerance is actually rare 84. For patients with SASEs who meet evidence-based guideline criteria for statin therapy, avoiding complete discontinuation of statin treatment is recommended whenever possible. In the SAMSON (Self-Assessment Method for Statin Side effects Or Nocebo) trial of patients who discontinued a statin due to SASEs, 90% of the adverse symptom effects experienced with drug therapy can be attributed to what is seen with a blinded placebo. 85

Although there is no universally accepted definition of statin intolerance, most experts recommend that patients are documented to have unacceptable muscle-related symptoms that resolve with discontinuation of therapy and recur with rechallenge on at least two (and preferably 3) statins, preferably ones that are metabolized by different pathways and have different lipophilicity/hydrophilicity, and one of which is prescribed at the lowest approved dose. Non-statin therapies can help manage dyslipidemia in such patients who have statin intolerance. 86

A trial of bempedoic acid + ezetimibe may be considered as first-line non-statin therapy in such patients. As an alternative, PCSK9 monoclonal antibodies ± ezetimibe may be considered depending on the patient’s clinical scenario. Inclisiran may also be considered especially in patients who are not compliant to treatment. 86

## Conclusions

This consensus statement emphasizes key recommendations on the use of non-statin therapies in the management of cardiovascular disease (CVD). We highlight the importance of identifying patients at extreme CV risk, which allows for targeted interventions and personalized treatments. We believe that statins will no longer stand alone in the battle against atherosclerosis. Furthermore, we propose considering initial combination therapy for individuals at very high and extreme risk, and suggest expanded use of bempedoic acid in patients who are still not at LDL-C goal on statin/ezetimibe combination especially in resource-limited settings as well as in statin-intolerant patients. These recommendations aim to optimize treatment strategies and enhance patient outcomes. However, further research and real-world data are necessary to validate and refine the implementation of these recommendations.

## Data Availability

The dataset supporting the results and conclusions of this article will be available from the corresponding author on request.

## Abbreviations

ACL: ATP citrate lyase
ACS: Acute coronary syndrome
ANGPTL3: Angiopoietin-like protein 3
ASCVD: Atherosclerotic cardiovascular disease
AUs: Agatston units
CABG: Coronary artery bypass surgery
CAC: Coronary artery calcium
CHD: Coronary heart disease
CHF: Congestive heart failure
CKD: Chronic kidney disease
CV: Cardiovascular
CVD: Cardiovascular diseases
DM: Diabetes mellitus
ESC: European Society of Cardiology
FDA: Food and Drug Administration
FH: Familial hypercholesterolemia
GBD: Global Burden of Disease
HDL: High-density lipoprotein
HeFH: Heterozygous familial hypercholesterolemia
HMG-CoA: Hydroxymethylglutaryl-coenzyme A
HoFH: Homozygous familial hypercholesterolemia
HTN: Hypertension
LDL-C: Low-density lipoprotein cholesterol
LLT: Lipid-lowering therapies
Lp (a): Lipoprotein (a)
MACE: Major adverse cardiovascular events
MI: Myocardial infarction
MTP: Microsomal triglyceride transfer protein
NPC1L1: Niemann–Pick C1-Like-1
PAD: Peripheral arterial disease
PCEs: Pooled cohort equations
PCI: Percutaneous coronary intervention
PCSK9: Proprotein convertase subtilisin/kexin type 9
RCTs: Randomized controlled trials
SASEs: Statin-associated side effects
siRNA: Small interfering RNA
TG: Triglyceride
TOD: Target organ damage

## References

[1] World Heart Report 2023|World Heart Federation. Accessed March 30, 2024. https://world-heart-federation.org/world-heart-report-2023/

[2] Vaduganathan M, Mensah GA, Turco JV, Fuster V, Roth GA (2022) The global burden of cardiovascular diseases and risk: a compass for future health. J Am Coll Cardiol 80(25):2361–2371. 10.1016/J.JACC.2022.11.005 36368511 10.1016/j.jacc.2022.11.005

[3] Ference BA, Ginsberg HN, Graham I et al (2017) Low-density lipoproteins cause atherosclerotic cardiovascular disease. 1. Evidence from genetic, epidemiologic, and clinical studies. A consensus statement from the European Atherosclerosis Society Consensus Panel. Eur Heart J 38(32):2459–2472 28444290 10.1093/eurheartj/ehx144 PMC5837225

[4] Naoumova RP, Marais AD, Mountney J et al (1996) Plasma mevalonic acid, an index of cholesterol synthesis in vivo, and responsiveness to HMG-CoA reductase inhibitors in familial hypercholesterolaemia. Atherosclerosis 119(2):203–213. 10.1016/0021-9150(95)05649-1 8808497 10.1016/0021-9150(95)05649-1

[5] Nguyen HT, Ha KPT, Nguyen AH, Nguyen TT, Lam HM (2021) Non-achievement of the low-density lipoprotein cholesterol goal in older patients with type 2 diabetes mellitus and a very high cardiovascular disease risk: a multicenter study in Vietnam. Ann Geriatr Med Res 25(4):278–285. 10.4235/AGMR.21.0099 34865341 10.4235/agmr.21.0099 PMC8749037

[6] Bello-Chavolla OY, Aguilar-Salinas CA (2019) Factors influencing achievement of low-density lipoprotein cholesterol goals in mexico: the international cholesterol management practice study. Rev Invest Clin 71(6):408–416. 10.24875/RIC.19003156 31823964 10.24875/RIC.19003156

[7] Butalia S, Chen G, Duan Q, Anderson TJ (2022) Care gaps in achieving cholesterol targets in people with diabetes: a population-based study in a universal health care setting. Diabetes Res Clin Pract. 10.1016/J.DIABRES.2021.109177 34923023 10.1016/j.diabres.2021.109177

[8] Barrios V, Pintó X, Escobar C, Varona JF, Gámez JM (2023) Real-world attainment of low-density lipoprotein cholesterol goals in patients at high risk of cardiovascular disease treated with high-intensity statins: The TERESA study. J Clin Med. 10.3390/JCM12093187 37176627 10.3390/jcm12093187 PMC10179558

[9] Sun L, Wolska A, Amar M, Zubirán R, Remaley AT (2023) Approach to the patient with a suboptimal statin response: causes and algorithm for clinical management. J Clin Endocrinol Metab 108(9):2424–2434. 10.1210/CLINEM/DGAD153 36929838 10.1210/clinem/dgad153 PMC10438872

[10] Carmena R, Roederer G, Mailloux H, Lussier-Cacan S, Davignon J (1993) The response to lovastatin treatment in patients with heterozygous familial hypercholesterolemia is modulated by apolipoprotein E polymorphism. Metab - Clin Exp 42(7):895–901. 10.1016/0026-0495(93)90066-W 8345800 10.1016/0026-0495(93)90066-w

[11] Bradley CK, Wang TY, Li S et al (2019) Patient-reported reasons for declining or discontinuing statin therapy: insights from the PALM registry. J Am Heart Assoc. 10.1161/JAHA.118.011765 30913959 10.1161/JAHA.118.011765 PMC6509731

[12] Bytyçi I, Penson PE, Mikhailidis DP et al (2022) Prevalence of statin intolerance: a meta-analysis. Eur Heart J 43(34):3213–3223. 10.1093/EURHEARTJ/EHAC015 35169843 10.1093/eurheartj/ehac015 PMC9757867

[13] Gao Y, Zhang B, Yang J (2022) Evinacumab for the treatment of homozygous familial hypercholesterolemia. Expert Rev Clin Pharmacol 15(2):139–145. 10.1080/17512433.2022.2047934 35220876 10.1080/17512433.2022.2047934

[14] Ray KK, Reeskamp LF, Laufs U et al (2022) Combination lipid-lowering therapy as first-line strategy in very high-risk patients. Eur Heart J 43(8):830–833. 10.1093/EURHEARTJ/EHAB718 34636884 10.1093/eurheartj/ehab718

[15] Rhee MY, Kim KJ, Kim SH et al (2019) Ezetimibe and Rosuvastatin combination treatment can reduce the dose of Rosuvastatin without compromising its lipid-lowering efficacy. Clin Ther 41(12):2571–2592. 10.1016/J.CLINTHERA.2019.10.010 31727361 10.1016/j.clinthera.2019.10.010

[16] Why Combination Lipid-Lowering Therapy Should be Considered Early in the Treatment of Elevated LDL-C For CV Risk Reduction - American College of Cardiology. Accessed March 30, 2024. https://www.acc.org/latest-in-cardiology/articles/2022/06/01/12/11/why-combination-lipid-lowering-therapy-should-be-considered

[17] El Etriby A, Bramlage P, El Nashar A, Brudi P (2013) The DYSlipidemia international study (DYSIS)-Egypt: a report on the prevalence of lipid abnormalities in Egyptian patients on chronic statin treatment. Egypt Hear J 65(3):223–232. 10.1016/J.EHJ.2013.05.003

[18] Ballantyne CM, Banka P, Mendez G et al (2023) Phase 2b randomized trial of the oral PCSK9 inhibitor MK-0616. J Am Coll Cardiol 81(16):1553–1564. 10.1016/J.JACC.2023.02.018 36889610 10.1016/j.jacc.2023.02.018

[19] Akoumianakis I, Zvintzou E, Kypreos K, Filippatos TD (2021) ANGPTL3 and apolipoprotein C-III as novel lipid-lowering targets. Curr Atheroscler Rep. 10.1007/S11883-021-00914-7 33694000 10.1007/s11883-021-00914-7

[20] Chamberlain AM, Gong Y, Shaw KMA et al (2019) PCSK9 inhibitor use in the real world: data from the national patient-centered research network. J Am Heart Assoc. 10.1161/JAHA 31020929 10.1161/JAHA.118.011246 PMC6512121

[21] Kazi DS, Lu CY, Lin GA et al (2017) Nationwide coverage and cost-sharing for PCSK9 inhibitors among medicare part D plans. JAMA Cardiol 2(10):1164. 10.1001/JAMACARDIO.2017.3051 28903137 10.1001/jamacardio.2017.3051 PMC5815006

[22] Kazi DS, Moran AE, Coxson PG et al (2016) Cost-effectiveness of PCSK9 inhibitor therapy in patients with heterozygous familial hypercholesterolemia or atherosclerotic cardiovascular disease. JAMA 316(7):743–753. 10.1001/JAMA.2016.11004 27533159 10.1001/jama.2016.11004

[23] Mortality and global health estimates. Accessed March 30, 2024. https://www.who.int/data/gho/data/themes/mortality-and-global-health-estimates

[24] Reda A, Bendary A, Elbahry A et al (2021) Prevalence of atherosclerosis risk factors in Egyptian patients with acute coronary syndrome: final data of the nationwide cross-sectional “CardioRisk” project. J Public Health Africa 11(2):114–121. 10.4081/JPHIA.2020.1368 10.4081/jphia.2020.1368 PMC7893316 33623654

[25] Reda A, Soliman M, El Kersh A et al (2019) The pattern of risk-factor profile in Egyptian patients with acute coronary syndrome: phase II of the Egyptian cross-sectional CardioRisk project. Cardiovasc J Afr 30(2):87–94. 10.5830/CVJA-2018-074 30720847 10.5830/CVJA-2018-074

[26] Roth GA, Mensah GA, Johnson CO et al (2020) Global burden of cardiovascular diseases and risk factors, 1990–2019: update from the GBD 2019 study. J Am Coll Cardiol 76(25):2982–3021. 10.1016/J.JACC.2020.11.010 33309175 10.1016/j.jacc.2020.11.010 PMC7755038

[27] Ray KK, Haq I, Bilitou A et al (2023) Treatment gaps in the implementation of LDL cholesterol control among high- and very high-risk patients in Europe between 2020 and 2021: the multinational observational SANTORINI study. Lancet Reg Heal Eur. 10.1016/J.LANEPE.2023.100624 10.1016/j.lanepe.2023.100624 PMC10119631 37090089

[28] Ray KK, Molemans B, Marieke Schoonen W et al (2021) EU-wide cross-sectional observational study of lipid-modifying therapy use in secondary and primary care: the DA VINCI study. Eur J Prev Cardiol 28(11):1279–1289. 10.1093/EURJPC/ZWAA047 33580789 10.1093/eurjpc/zwaa047

[29] Lloyd-Jones DM, Morris PB, Ballantyne CM et al (2022) ACC expert consensus decision pathway on the role of nonstatin therapies for LDL-cholesterol lowering in the management of atherosclerotic cardiovascular disease risk: a report of the American college of cardiology solution set oversight committee. J Am Coll Cardiol 80(14):1366–1418 36031461 10.1016/j.jacc.2022.07.006

[30] Nissen SE, Lincoff AM, Brennan D et al (2023) Bempedoic acid and cardiovascular outcomes in statin-intolerant patients. N Engl J Med 388(15):1353–1364. 10.1056/NEJMOA2215024/SUPPL_FILE/NEJMOA2215024_DATA-SHARING.PDF 36876740 10.1056/NEJMoa2215024

[31] Schwartz GG, Steg PG, Szarek M et al (2018) Alirocumab and cardiovascular outcomes after acute coronary syndrome. N Engl J Med 379(22):2097–2107 30403574 10.1056/NEJMoa1801174

[32] Laufs U, Ballantyne CM, Banach M et al (2022) Efficacy and safety of bempedoic acid in patients not receiving statins in phase 3 clinical trials. J Clin Lipidol 16(3):286–297. 10.1016/J.JACL.2022.03.001 35346603 10.1016/j.jacl.2022.03.001

[33] Ballantyne CM, Laufs U, Ray KK et al (2020) Bempedoic acid plus ezetimibe fixed-dose combination in patients with hypercholesterolemia and high CVD risk treated with maximally tolerated statin therapy. Eur J Prev Cardiol 27(6):593–603. 10.1177/2047487319864671 31357887 10.1177/2047487319864671 PMC7153222

[34] Fitzgerald K, White S, Borodovsky A et al (2017) A highly durable RNAi therapeutic inhibitor of PCSK9. N Engl J Med 376(1):41–51. 10.1056/NEJMOA1609243/SUPPL_FILE/NEJMOA1609243_DISCLOSURES.PDF 27959715 10.1056/NEJMoa1609243 PMC5778873

[35] Raal FJ, Kallend D, Ray KK et al (2020) Inclisiran for the treatment of heterozygous familial hypercholesterolemia. N Engl J Med 382(16):1520–1530. 10.1056/NEJMOA1913805 32197277 10.1056/NEJMoa1913805

[36] Ray KK, Wright RS, Kallend D et al (2020) Two phase 3 trials of inclisiran in patients with elevated LDL cholesterol. N Engl J Med 382(16):1507–1519. 10.1056/NEJMOA1912387/SUPPL_FILE/NEJMOA1912387_DATA-SHARING.PDF 32187462 10.1056/NEJMoa1912387

[37] Ray KK, Troquay RPT, Visseren FLJ et al (2023) Long-term efficacy and safety of inclisiran in patients with high cardiovascular risk and elevated LDL cholesterol (ORION-3): results from the 4-year open-label extension of the ORION-1 trial. Lancet Diabetes Endocrinol 11(2):109–119. 10.1016/S2213-8587(22)00353-9 36620965 10.1016/S2213-8587(22)00353-9

[38] Ray KK, Bays HE, Catapano AL et al (2019) Safety and efficacy of bempedoic acid to reduce LDL cholesterol. N Engl J Med 380(11):1022–1032. 10.1056/NEJMOA1803917 30865796 10.1056/NEJMoa1803917

[39] Rifkind BM (1984) Lipid research clinics coronary primary prevention trial: results and implications. Am J Cardiol 54(5):30–34. 10.1016/0002-9149(84)90854-3 10.1016/0002-9149(84)90854-3 6382999

[40] Bruckert E, Giral P, Tellier P (2003) Perspectives in cholesterol-lowering therapy the role of ezetimibe, a new selective inhibitor of intestinal cholesterol absorption. Published online. 10.1161/01.CIR.0000072345.98581.24 10.1161/01.CIR.0000072345.98581.24 12835406

[41] Knopp RH, Gitter H, Truitt T et al (2003) Effects of ezetimibe, a new cholesterol absorption inhibitor, on plasma lipids in patients with primary hypercholesterolemia. Eur Heart J 24(8):729–741. 10.1016/S0195-668X(02)00807-2 12713767 10.1016/s0195-668x(02)00807-2

[42] Cannon CP, Blazing MA, Giugliano RP et al (2015) Ezetimibe added to statin therapy after acute coronary syndromes. N Engl J Med 372(25):2387–2397. 10.1056/NEJMOA1410489/SUPPL_FILE/NEJMOA1410489_DISCLOSURES.PDF 26039521 10.1056/NEJMoa1410489

[43] Pradhan A, Bhandari M, Sethi R (2020) Ezetimibe and improving cardiovascular outcomes: current evidence and perspectives. Cardiol Res Pract. 10.1155/2020/9815016 32670636 10.1155/2020/9815016 PMC7338976

[44] Baigent C, Landray MJ, Reith C et al (2011) The effects of lowering LDL cholesterol with simvastatin plus ezetimibe in patients with chronic kidney disease (Study of Heart and Renal Protection): a randomised placebo-controlled trial. Lancet (London, England) 377(9784):2181–2192. 10.1016/S0140-6736(11)60739-3 21663949 10.1016/S0140-6736(11)60739-3 PMC3145073

[45] Kim BK, Hong SJ, Lee YJ et al (2022) Long-term efficacy and safety of moderate-intensity statin with ezetimibe combination therapy versus high-intensity statin monotherapy in patients with atherosclerotic cardiovascular disease (RACING): a randomised, open-label, non-inferiority trial. Lancet (London, England) 400(10349):380–390. 10.1016/S0140-6736(22)00916-3 35863366 10.1016/S0140-6736(22)00916-3

[46] Shapiro MD, Tavori H, Fazio S (2018) PCSK9: from basic science discoveries to clinical trials. Circ Res 122(10):1420–1438. 10.1161/CIRCRESAHA.118.311227 29748367 10.1161/CIRCRESAHA.118.311227 PMC5976255

[47] Sabatine MS, Giugliano RP, Keech AC et al (2017) Evolocumab and clinical outcomes in patients with cardiovascular disease. N Engl J Med 376(18):1713–1722. 10.1056/NEJMOA1615664/SUPPL_FILE/NEJMOA1615664_DISCLOSURES.PDF 28304224 10.1056/NEJMoa1615664

[48] Singh A, Cho LS (2024) Nonstatin therapy to reduce low-density lipoprotein cholesterol and improve cardiovascular outcomes. Cleve Clin J Med 91(1):53–63. 10.3949/CCJM.91A.23058 38167398 10.3949/ccjm.91a.23058

[49] Visseren FLJ, MacH F, Smulders YM et al (2021) ESC Guidelines on cardiovascular disease prevention in clinical practice. Eur Heart J. 10.1093/eurheartj/ehab484 34458905 10.1093/eurheartj/ehab484

[50] Marx N, Federici M, Schütt K et al (2023) 2023 ESC Guidelines for the management of cardiovascular disease in patients with diabetes: Developed by the task force on the management of cardiovascular disease in patients with diabetes of the European Society of Cardiology (ESC). Eur Heart J 44(39):4043–4140. 10.1093/EURHEARTJ/EHAD192 37622663 10.1093/eurheartj/ehad192

[51] Mach F, Baigent C, Catapano AL et al (2020) 2019 ESC/EAS Guidelines for the management of dyslipidaemias: lipid modification to reduce cardiovascular risk: the task force for the management of dyslipidaemias of the European Society of Cardiology (ESC) and European Atherosclerosis Society (EAS). Eur Heart J 41(1):111–188. 10.1093/EURHEARTJ/EHZ455 31504418 10.1093/eurheartj/ehz455

[52] Newman CB, Preiss D, Tobert JA et al (2019) Statin safety and associated adverse events: a scientific statement from the American heart association. Arterioscler Thromb Vasc Biol 39(2):e38–e81. 10.1161/ATV.0000000000000073 30580575 10.1161/ATV.0000000000000073

[53] Xie C, Zhou ZS, Li N, Bian Y, Wang YJ, Wang LJ, Li BL, Song BL et al (2012) Ezetimibe blocks the internalization of NPC1L1 and cholesterol in mouse small intestine. J Lipid Res 53(10):2092–2101. 10.1194/jlr.M027359 22811412 10.1194/jlr.M027359 PMC3435542

[54] Murphy SA, Cannon CP, Blazing MA et al (2016) Reduction in total cardiovascular events with ezetimibe/simvastatin post-acute coronary syndrome: the IMPROVE-IT trial. J Am Coll Cardiol 67(4):353–361 26821621 10.1016/j.jacc.2015.10.077

[55] Hammersley D, Signy M (2017) Ezetimibe: an update on its clinical usefulness in specific patient groups. Ther Adv Chronic Dis 8(1):4–11 28203346 10.1177/2040622316672544 PMC5298356

[56] Roth EM, Davidson MH. PCSK9 inhibitors: mechanism of action, efficacy, and safety. *Rev Cardiovasc Med*. 2018, 19. 10.3909/ricm19S1S0002 30207556

[57] Sabatine MS, Giugliano RP, Keech AC et al (2017) Evolocumab and clinical outcomes in patients with cardiovascular disease. N Engl J Med 376(18):1713–1722 28304224 10.1056/NEJMoa1615664

[58] Wright RS, Ray KK, Raal FJ et al (2021) Pooled patient-level analysis of inclisiran trials in patients with familial hypercholesterolemia or atherosclerosis. J Am Coll Cardiol 77(9):1182–1193. 10.1016/j.jacc.2020.12.058 33663735 10.1016/j.jacc.2020.12.058

[59] Albosta MS, Grant JK, Taub P, Blumenthal RS, Martin SS, Michos ED (2023) Inclisiran: a new strategy for LDL-C lowering and prevention of atherosclerotic cardiovascular disease. Vasc Health Risk Manag 19:421–431. 10.2147/VHRM.S338424 37434791 10.2147/VHRM.S338424 PMC10332363

[60] Highlights of Prescribing Information. Accessed April 2, 2024. www.fda.gov/ medwatch.

[61] Bempedoic Acid and Ezetimibe Combination Approved by FDA for Lowering LDL-C. Accessed August 18, 2024. https://www.hcplive.com/view/nexlizet-bempec-acid-ezetimibe-combination-approved-fda-cholesterol-esperion

[62] Cho L (2023) Bempedoic acid and cardiovascular outcomes in statin intolerant patients at high cardiovascular risk: clear outcome. J Clin Lipidol 17(4):e62

[63] Danis RP, Gangaputra S, Hubbard L et al (2010) Effects of medical therapies on retinopathy progression in Type 2 diabetes. N Engl J Med 363(3):233–244. 10.1056/NEJMOA1001288 20587587 10.1056/NEJMoa1001288 PMC4026164

[64] Lent-Schochet D, Jialal I. Antilipemic Agent Bile Acid Sequestrants. *StatPearls*. Published online January 23, 2023. Accessed April 2, 2024. https://www.ncbi.nlm.nih.gov/books/NBK549906/31751096

[65] Raal FJ, Rosenson RS, Reeskamp LF et al (2020) Evinacumab for homozygous familial hypercholesterolemia. N Engl J Med 383(8):711–720. 10.1056/NEJMOA2004215 32813947 10.1056/NEJMoa2004215

[66] Patel N, Parmar M, Patel P. Evinacumab. *StatPearls*. Published online October 28, 2023. Accessed April 2, 2024. https://www.ncbi.nlm.nih.gov/books/NBK597342/

[67] Blom DJ, Raal FJ, Santos RD, Marais AD (2019) Lomitapide and mipomersen-inhibiting microsomal triglyceride transfer protein (MTP) and apoB100 synthesis. Curr Atheroscler Rep 21(12):1–10. 10.1007/S11883-019-0809-3 31741187 10.1007/s11883-019-0809-3

[68] Nugent AK, Gray JV, Gorby LK, Moriarty PM (2020) Lipoprotein apheresis: first FDA indicated treatment for elevated lipoprotein (a). J Clin Cardiol 1(1):16–21

[69] Kayikcioglu M (2021) LDL apheresis and Lp (a) apheresis: a clinician’s perspective. Curr Atheroscler Rep 23(4):1–12. 10.1007/S11883-021-00911-W 10.1007/s11883-021-00911-w PMC7886643 33594522

[70] Stone NJ, Robinson JG, Lichtenstein AH et al (2014) 2013 ACC/AHA guideline on the treatment of blood cholesterol to reduce atherosclerotic cardiovascular risk in adults: a report of the american college of cardiology/american heart association task force on practice guidelines. Circulation 129(25 SUPPL. 1):1–45. 10.1161/01.CIR.0000437738.63853.7A/-/DC1 10.1161/01.cir.0000437738.63853.7a 24222016

[71] Tavazzi L, Maggioni AP, Marchioli R, Barlera S, Franzosi MG, Latini R, Lucci D, Nicolosi GL, Porcu M, Tognoni G (2008) Effect of rosuvastatin in patients with chronic heart failure (the GISSI-HF trial): a randomised, double-blind, placebo-controlled trial. Lancet (London, England) 372(9645):1231–1239. 10.1016/S0140-6736(08)61240-4 18757089 10.1016/S0140-6736(08)61240-4

[72] Kjekshus J, Apetrei E, Barrios V et al (2007) Rosuvastatin in older patients with systolic heart failure. N Engl J Med 357(22):2248–2261. 10.1056/NEJMOA0706201 17984166 10.1056/NEJMoa0706201

[73] Feinstein MJ, Jhund P, Kang J et al (2015) Do statins reduce the risk of myocardial infarction in patients with heart failure? A pooled individual-level reanalysis of CORONA and GISSI-HF. Eur J Heart Fail 17(4):434–441. 10.1002/EJHF.247 25684642 10.1002/ejhf.247

[74] Ray KK, Landmesser U, Leiter LA et al (2017) Inclisiran in patients at high cardiovascular risk with elevated LDL cholesterol. N Engl J Med 376(15):1430–1440. 10.1056/NEJMOA1615758/SUPPL_FILE/NEJMOA1615758_DISCLOSURES.PDF 28306389 10.1056/NEJMoa1615758

[75] FDA requests removal of strongest warning against using cholesterol-lowering statins during pregnancy; still advises most pregnant patients should stop taking statins | FDA. Accessed April 2, 2024. https://www.fda.gov/drugs/fda-drug-safety-podcasts/fda-requests-removal-strongest-warning-against-using-cholesterol-lowering-statins-during-pregnancy

[76] Shepherd J, Blauw GJ, Murphy MB et al (2002) Pravastatin in elderly individuals at risk of vascular disease (PROSPER): a randomised controlled trial. Lancet 360(9346):1623–1630. 10.1016/S0140-6736(02)11600-X 12457784 10.1016/s0140-6736(02)11600-x

[77] Schreuder MM, Hamkour S, Siegers KE et al (2023) LDL cholesterol targets rarely achieved in familial hypercholesterolemia patients: a sex and gender-specific analysis. Atherosclerosis 384:117117. 10.1016/J.ATHEROSCLEROSIS.2023.03.022 37080805 10.1016/j.atherosclerosis.2023.03.022

[78] Raal FJ, Stein EA, Dufour R et al (2015) PCSK9 inhibition with evolocumab (AMG 145) in heterozygous familial hypercholesterolaemia (RUTHERFORD-2): a randomised, double-blind, placebo-controlled trial. Lancet 385(9965):331–340. 10.1016/S0140-6736(14)61399-4 25282519 10.1016/S0140-6736(14)61399-4

[79] Kastelein JJP, Ginsberg HN, Langslet G et al (2015) ODYSSEY FH I and FH II: 78 week results with alirocumab treatment in 735 patients with heterozygous familial hypercholesterolaemia. Eur Heart J 36(43):2996–3003. 10.1093/EURHEARTJ/EHV370 26330422 10.1093/eurheartj/ehv370 PMC4644253

[80] Raal FJ, Honarpour N, Blom DJ et al (2015) Inhibition of PCSK9 with evolocumab in homozygous familial hypercholesterolaemia (TESLA Part B): a randomised, double-blind, placebo-controlled trial. Lancet (London, England) 385(9965):341–350. 10.1016/S0140-6736(14)61374-X 25282520 10.1016/S0140-6736(14)61374-X

[81] Raal FJ, Hovingh GK, Blom D et al (2017) Long-term treatment with evolocumab added to conventional drug therapy, with or without apheresis, in patients with homozygous familial hypercholesterolaemia: an interim subset analysis of the open-label TAUSSIG study. Lancet Diabetes Endocrinol 5(4):280–290. 10.1016/S2213-8587(17)30044-X 28215937 10.1016/S2213-8587(17)30044-X

[82] Stein EA, Honarpour N, Wasserman SM, Xu F, Scott R, Raal FJ (2013) Effect of the proprotein convertase subtilisin/kexin 9 monoclonal antibody, AMG 145, in homozygous familial hypercholesterolemia. Circulation 128(19):2113–2120. 10.1161/CIRCULATIONAHA.113.004678/-/DC1 24014831 10.1161/CIRCULATIONAHA.113.004678

[83] Blom DJ, Harada-Shiba M, Rubba P et al (2020) Efficacy and Safety of alirocumab in adults with homozygous familial Hypercholesterolemia: the ODYSSEY HoFH trial. J Am Coll Cardiol 76(2):131–142. 10.1016/J.JACC.2020.05.027 32646561 10.1016/j.jacc.2020.05.027

[84] Cheeley MK, Clegg K, Lockridge C, Schubert TJ, Jones LK (2023) Statin Intolerance: an overview of US and international guidance. Curr Atheroscler Rep 25(8):517–526. 10.1007/S11883-023-01124-Z/TABLES/2 37410332 10.1007/s11883-023-01124-z PMC10412662

[85] Nelson AJ, Pagidipati NJ, Granger CB (2021) The SAMSON trial: using a placebo to improve medication tolerability. Eur Hear J - Cardiovasc Pharmacother 7(3):e13–e13. 10.1093/EHJCVP/PVAB017 10.1093/ehjcvp/pvab017 33638980

[86] NLA 2022 Definition of Statin Intolerance - American College of Cardiology. Accessed April 2, 2024. https://www.acc.org/latest-in-cardiology/articles/2022/08/08/12/27/nla-2022-definition-of-statin-intolerance

